# Infant With Recurrent Vomiting and Poor Weight Gain Secondary to an Aberrant Subclavian Artery

**DOI:** 10.7759/cureus.36856

**Published:** 2023-03-29

**Authors:** John Dugas, Amber Vozar, Seth J Deskins, Sharda Udassi

**Affiliations:** 1 Pediatrics, West Virginia University, Morgantown, USA; 2 Internal Medicine, Pediatrics, West Virginia University (WVU) Medicine, Morgantown, USA

**Keywords:** emesis, esophageal compression, abnormal branching of the subclavian artery, failure to thrive, inpatient pediatrics

## Abstract

Failure to thrive (FTT) is a term commonly used to characterize slower-than-expected weight gain. While inadequate caloric intake is the predominant cause, failure to thrive is a manifestation of undernutrition often resulting from the interplay of multiple etiologies. This case highlights the diagnosis and management of an infant who presented with recurrent large-volume emesis and poor weight gain secondary to esophageal compression from an aberrant right subclavian artery (ARSA).

## Introduction

Failure to thrive (FTT) is a general term used to diagnose pediatric patients who weigh less than the fifth percentile for sex and age or who have a sustained downward growth velocity crossing two major percentiles on a standardized growth curve [1]. Untreated malnutrition resulting in FTT increases the risk for complications of acute illness and can lead to long-term psychosocial impairment and developmental delay [2]. The American Academy of Family Physicians (AAFP) uses the following three categories to classify the pathogenesis of FTT: inadequate caloric intake, inadequate absorption of nutrients, or excessive caloric expenditure [1]. One of the most common causes of insufficient caloric intake in infants is regurgitation from gastroesophageal reflux (GER) [2]. Though infantile reflux is non-pathologic, it is critical to pursue further evaluation of recurrent vomiting, which hinders growth and development, as esophageal compression from anatomic abnormalities can have a clinical manifestation resembling GER disease [3]. Here, we present a case of an aberrant right subclavian artery (ARSA) causing esophageal compression and recurrent emesis, ultimately leading to FTT.

## Case presentation

**Figure 1:**
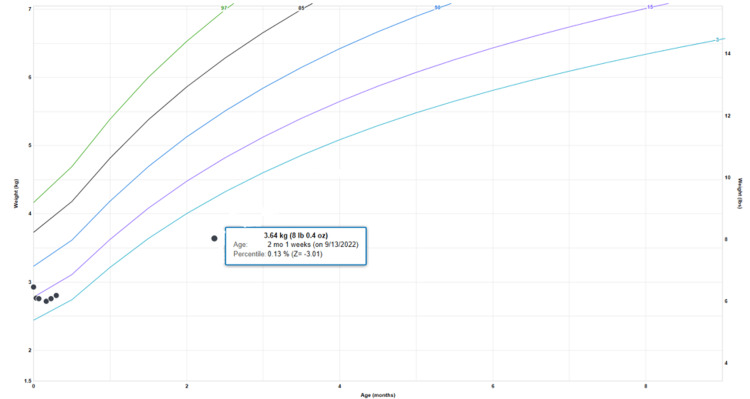
The World Health Organization's weight-for-age chart for girls (0 to 2 years) shows our patient's <1st percentile in weight-for-age on presentation

A 72-day-old female born late pre-term at 35 weeks and five days was admitted to our facility from her pediatrician’s office for persistent emesis and workup of poor weight gain, previously known as FTT. Her birth weight was 2.93 kg, and her lowest weight was 2.72 kg (7% down from birth weight) at five days of age. Though she surpassed her birth weight at 14 days of life, she weighed only 3.64 kg (less than the first percentile on the weight-for-age growth chart) at admission on day of life 72 (Figure 1).

The patient’s mother reported recurrent large volume non-bloody, non-bilious emesis since birth, not associated with feeds. She tried multiple different formulas, bottle systems, and nipples as well as feeding smaller volumes more frequently (down to 1 oz every hour), without symptomatic improvement. She also kept the patient upright for at least 30 minutes following feeds without symptomatic improvement. The patient had a pyloric ultrasound (US) completed on day of life 51, which was normal.

**Figure 2:**
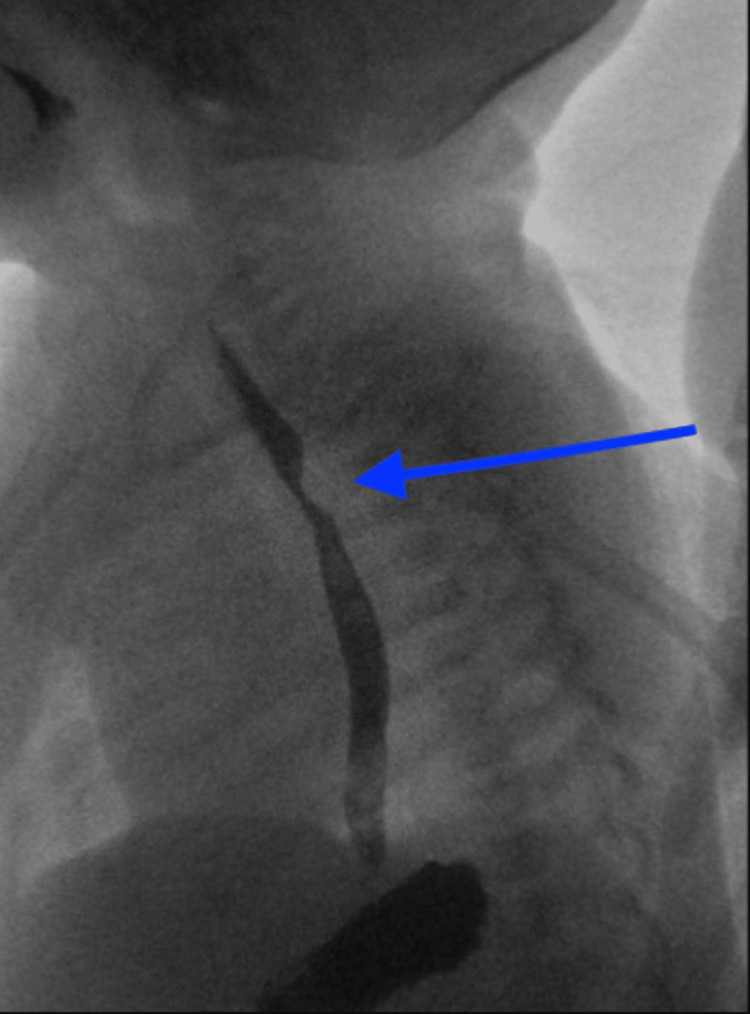
Fluoroscopy upper GI radiograph depicting a small posterior indentation of the upper esophagus, concerning for a vascular ring or sling GI: Gastrointestinal

On admission, laboratory work-up including a basic metabolic panel, complete blood count, and hepatic function panel were within normal limits. An upper gastrointestinal (GI) barium study was performed, which revealed a posterior indentation of the esophagus, concerning for a vascular ring or sling (Figure 2).

**Figure 3:**
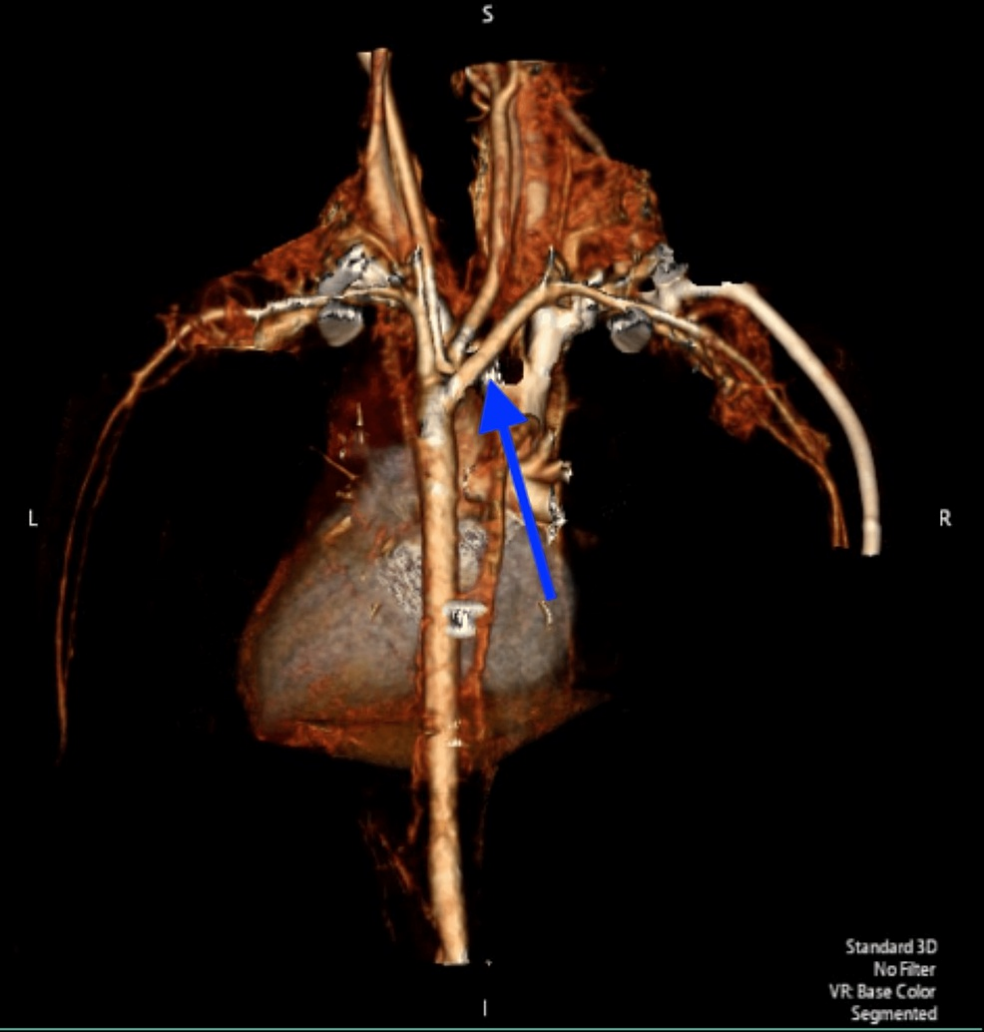
The 3D CTA shows a posterior view of the left-sided aortic arch with a common origin for right and left common carotid arteries as well as an ARSA arising directly from the aortic arch. 3D: Three dimensional, CTA: Computed tomography angiography, ARSA: Aberrant right subclavian artery

An echocardiogram and computed tomography angiography (CTA) (Figure 3) was subsequently performed, demonstrating a left aortic arch with an ARSA causing compression of the posterior esophagus.

A multidisciplinary conference with pediatric cardiology, gastroenterology, and cardiothoracic surgery discussed the patient’s case. The consensus was for surgical reimplantation, and the patient underwent successful reimplantation of the right subclavian artery into the right carotid artery. Her postoperative course was complicated by a large circumferential pericardial effusion requiring drain placement. After drain removal and effusion resolution, her emesis and weight trajectory significantly improved. She has gained an average of 26 grams per day after surgery and no longer experiences emesis with feeds.

## Discussion

Poor weight gain is most commonly a result of inadequate caloric intake. This is usually secondary to inorganic factors, such as incorrect formula preparation or neglect [1]. A thorough history can reveal more organic causes, including GER. Initial management of GER includes thickening of feeds, reassurance, and upright position after feeds. In the case of no improvement and signs of poor weight gain or respiratory symptoms, it is essential to consider other diagnoses and obtain an upper GI examination to evaluate the structure [2]. This study can identify foreign bodies or esophageal strictures that can cause regurgitation of feeds. One rare etiology of an esophageal stricture found in 0.5% to 1.8% of the population is an ARSA [3].

This anatomic phenomenon is also known as *dysphagia lusoria *and was first reported after a patient with longstanding dysphagia in 1787 had an autopsy that revealed a right subclavian artery originating from her aortic arch that compressed her esophagus [4]. Normally, the aortic arch has three branches: the innominate (brachiocephalic trunk), the left common carotid, and the left subclavian arteries. The innominate artery bifurcates into the right subclavian and right common carotid. In contrast in an ARSA, the aberrant vessel originates as the fourth branch that then is required to cross the midline posterior to the esophagus and trachea. This is problematic because, while transversing to the right arm, the vessel has the potential to compress the esophagus and trachea, leading to the sequela of dysphagia lusoria. 

The average age of symptom onset from ARSA is 50 years, and the most common symptoms are dysphagia, dyspnea, and retrosternal pain [5]. Symptoms can also be seen in infants and children; however, they present with more signs of tracheal obstruction rather than esophageal. A study by van Son et al. found that 86% of infant patients with ARSA had symptoms of recurrent respiratory infections or stridor [6]. Wheezing and cyanosis can also be seen in this population, and some patients have been mistaken for uncontrolled asthmatics; however, their symptoms do not improve with bronchodilator therapy [7]. One proposed explanation for respiratory symptoms in children is their less rigid trachea, which is more compressible from the esophagus secondary to ARSA [8]. 

A barium swallow is the best initial diagnostic test when there is a concern for an esophageal obstruction, such as an ARSA [9]. The imaging will show an oblique compression of the esophagus. An echocardiogram should also be performed for a detailed evaluation of intracardiac anatomy and function. A CTA is required to confirm the diagnosis and also to aid in surgical planning [10].

Even though our patient did not have the classic symptoms associated with infantile dysphagia lusoria, the decision was made to perform surgery due to severe poor weight gain in a setting of significant esophageal compression. Our patient underwent a median thoracotomy with reimplantation of the right subclavian artery into her right carotid artery. While various surgical approaches have proved successful, the technique is based on the surgeon's preference and the patient's presenting anatomy [10]. There are also cases in the literature where family members or providers decide against surgery. One case report discusses an infant diagnosed with ARSA who had frequent spit-ups and a respiratory symptom of inspiratory stridor. However, this patient was growing appropriately, and the family chose to treat symptomatically [11]. Ultimately, the decision for surgical intervention should be based on the individual patient and their clinical presentation.

## Conclusions

Poor weight gain is most likely caused by inorganic causes related to insufficient caloric intake; however, it is crucial to evaluate organic causes when inorganic etiologies have been ruled out. Although rare, an ARSA is an aortic arch abnormality that can cause GER and poor weight gain in infants. Most pediatric cases of ARSA typically manifest with respiratory symptoms, such as stridor or audible wheezing, whereas adults who present later in life have more gastrointestinal symptoms, like dysphagia. A barium study can suggest ARSA; however, CTA is the gold standard diagnostic test. Early detection is imperative and requires a high level of clinical suspicion for prompt treatment of ARSA. Overall, ARSA is a rare cause of poor weight gain but should be considered when other more common etiologies have been eliminated.
